# A Stretchable Radio-Frequency Strain Sensor Using Screen Printing Technology

**DOI:** 10.3390/s16111839

**Published:** 2016-11-02

**Authors:** Heijun Jeong, Sungjoon Lim

**Affiliations:** School of Electrical and Electronics Engineering, College of Engineering, Chung-Ang University, 221 Heukseok-dong, Dongjak-gu, Seoul 156-756, Korea; jhijun@naver.com

**Keywords:** stretchable dielectric material, stretchable conductive ink, PDMS, stretchable RF resonator

## Abstract

In this paper, we propose a stretchable radio-frequency (RF) strain sensor fabricated with screen printing technology. The RF sensor is designed using a half-wavelength patch that resonates at 3.7 GHz. The resonant frequency is determined by the length of the patch, and it therefore changes when the patch is stretched. Polydimethylsiloxane (PDMS) is used to create the substrate, because of its stretchable and screen-printable surface. In addition, Dupont PE872 (Dupont, NC, American) silver conductive ink is used to create the stretchable conductive patterns. The sensor performance is demonstrated both with full-wave simulations and with measurements carried out on a fabricated sample. When the length of the patch sensor is increased by a 7.8% stretch, the resonant frequency decreases from 3.7 GHz to 3.43 GHz, evidencing a sensitivity of 3.43 × 10^7^ Hz/%. Stretching the patch along its width does not change the resonant frequency.

## 1. Introduction

Strain sensors detect deformation and perform measurements when force is applied on them. Semiconductor-based sensors have a higher sensitivity and are lower in price than nanomaterial-based sensors [1,2]. Nevertheless, semiconductor-based sensors have the drawback of low resolution and they cannot be embedded in structural materials [3,4]. In addition, they can measure only specific directions.

Due to high demands on wearable and bio technologies, the importance of nanomaterial-based strain sensors is increasing. In addition, soft and flexible materials such as polydimethylsiloxane (PDMS) and Eco-flex have been widely studied in strain sensors based on nanomaterials (e.g., nanoparticles, nanowires, nanotubes) [5,6,7,8]. For instance, stretchable strain sensors are printed using silver nanowires [9]. A high-sensitivity strain sensor is fabricated using a single wire of gold nanoparticles [10]. A flexible self-repairing ground reaction sensor is developed using liquid metals and PDMS [11]. A highly stretchable strain sensor is proposed using a carbon nanotubes and Ecoflex nanocomposite [12]. However, these devices are difficult to fabricate. For example, the fabrication of microfluidic channels on an elastomer substrate requires, as processing steps, spin-coating the photoresist, exposure to UV light, patterning, and deposition [13,14]. Furthermore, the number of usable types of substrate is limited.

In this study, we use a screen printing method on an elastomer for radio-frequency (RF) strain sensor applications. Screen printing technology is a good candidate for sensor applications because of its simple fabrication and low-cost mass production [15,16]. In order to apply screen printing technology to stretchable electronics, stretchable conductive inks must be screen printed on stretchable dielectric materials. However, the feasibility of stretchable inks and dielectric materials has not been investigated for RF applications. To obtain stretchable characteristics, we used a polydimethylsiloxane (PDMS) substrate, which is easy to manufacture using simple processes. PDMS has favorable dielectric properties, and good flexibility and restoring capabilities [17,18]. Stretchable silver conductive ink (Dupont PE872) was used for screen printing. PE872 shows good conductivity and high durability, even after being washed with water. The screen printing method was used to design a patch on top of the PDMS; the ground plane was obtained in the same way. The device was then cured to improve conductivity. In this paper, we proposed a rectangular patch resonator for a strain sensor. We will investigate the relation between the resonant frequency and the patch length change through simulation and measurement.

## 2. Strain Sensor Design

**Figure 1:**
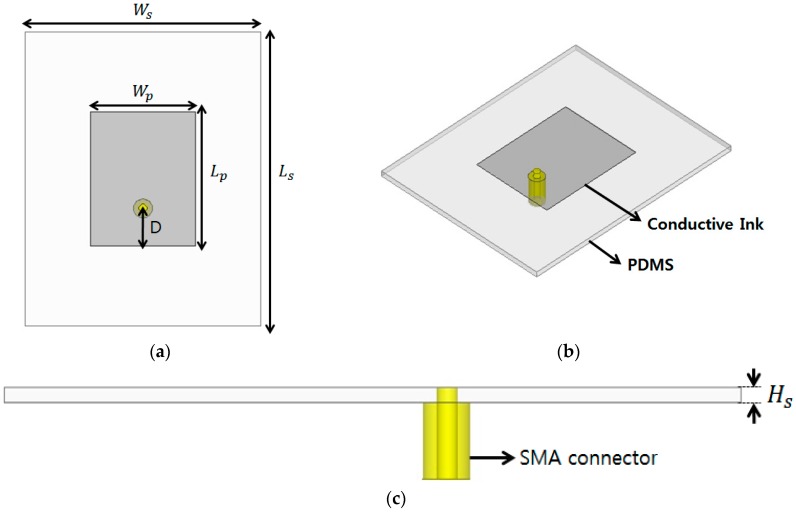
Geometry of the proposed radio-frequency (RF) strain sensor: (**a**) top view, (**b**) perspective view, and (**c**) side view. $W_{p}$ = 17 mm, $L_{p}$ = 23 mm, $W_{s}$ = 40 mm, $L_{s}$ = 50 mm, D = 6.5 mm, and $H_{s}$ = 1 mm.

The proposed strain sensor was designed based on a rectangular patch resonator. Rectangular patches are widely used for RF resonators and resonator-based components, because of their simple design and easy fabrication. In this work, the conductive patterns were created using screen printing technology, and the surface resistance of the conductive pattern was determined based on the width-to-length ratio. The surface resistance of the stretchable silver ink is 0.65 Ω and rectangular patch is 14 Ω. The lower surface resistance of the rectangular patch resonator is achieved due to lower width-to-length ratio. Figure 1 shows the geometry of the proposed patch resonator. A coaxial feeding line was employed (rather than microstrip feeding lines), so that the feeding line was not affected when the patch was stretched.

**Figure 2:**
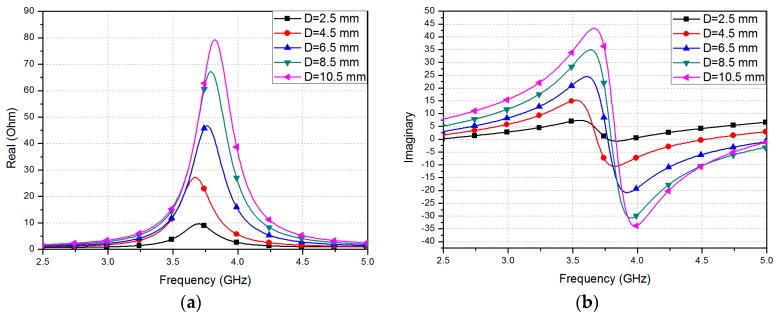
Simulated input impedance at different location of D; (**a**) real part and (**b**) imaginary part.

**Figure 3:**
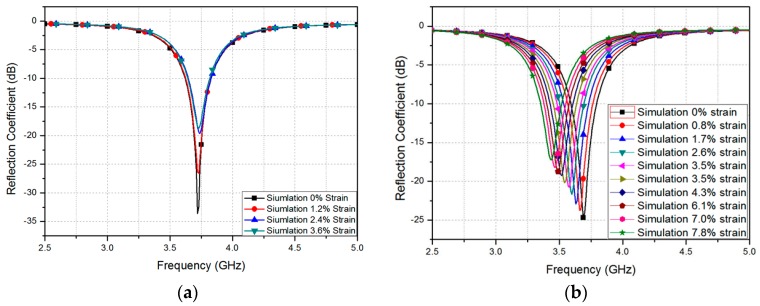
Simulated reflection coefficients at different patch (**a**) widths and (**b**) lengths.

As mentioned, PDMS—a stretchable dielectric material—was used as substrate for the patch resonator. Its dielectric constant (*ε_r_*) and loss tangent were determined using a T-resonator method [19,20]. The resonant frequency of the rectangular patch can be calculated as follows [21,22]:

(1) $$f_{0}=\frac{c}{2\sqrt{\varepsilon_{\mathrm{eff}}}\left\{L_{p}+0.824H_{s}\left[\frac{\left(\varepsilon_{\mathrm{eff}}+0.3\right)\left(\frac{W_{p}}{H_{s}}+0.264\right)}{\left(\varepsilon_{\mathrm{eff}}-0.258\right)\left(\frac{W_{p}}{H_{s}}+0.8\right)}\right]\right\}}$$

(2) $$\varepsilon_{\mathrm{eff}}=\frac{\varepsilon_{r}+1}{2}+\frac{\varepsilon_{r}-1}{2}\left[\frac{1}{\sqrt{1+12\left(\frac{h_{s}}{W_{p}}\right)}}\right]$$

where *ε*_eff_ is the effective dielectric constant resulting from the fringe fields and H_s_ is the thickness of the substrate. The values of *ε_r_* and loss tangent of the PDMS were determined to be 3 and 0.02, respectively. In order to characterize dielectric constants and loss tangent of the PDMS, we used T-resonator technique, as introduced in [19,20]. The width (*W_p_*) and length (*L_p_*) of the rectangular patch were therefore set at 17 mm and 23 mm, respectively, to allow the device to resonate at 3.7 GHz. Figure 2a,b shows real and imaginary parts of the input impedance at different locations of D, respectively. It is observed from Figure 2 that both impedance and resonant frequency decrease as D increases. Therefore, the location of the coaxial feeding (D) is determined to be 6.5 mm to achieve 50-Ω impedance matching. The ANSYS high frequency structure simulator (HFSS) was used for full-wave simulation. A SubMiniature version A (SMA) connector was included in the design for this simulation, as shown in Figure 1. Given that the resonant frequency is dependent on the length *L_p_* of the patch, we expect the resonant frequency to decrease when the patch is stretched. Figure 3a,b shows the simulated reflection coefficients for different values of *W_p_* and *L_p_*, respectively. Initially, the unstretched patch sensor resonates at 3.7 GHz, with a −25 dB reflection coefficient. As shown in Figure 3a, the resonant frequency does not change while *W_p_* is varied [23]. However, as shown in Figure 3b, the resonant frequency decreases from 3.7 GHz to 3.43 GHz when the patch is stretched by 7.8% along its length. Therefore, the proposed patch resonator can be used as a strain sensor by detecting changes in its resonant frequency. In order to measure the level of stretchability, strain is defined as

(3) $$Strain=\frac{\Delta L}{L_{0}}\times 100(\%)$$

## 3. Strain Sensor Fabrication

### 3.1. PDMS Fabrication

**Figure 4:**
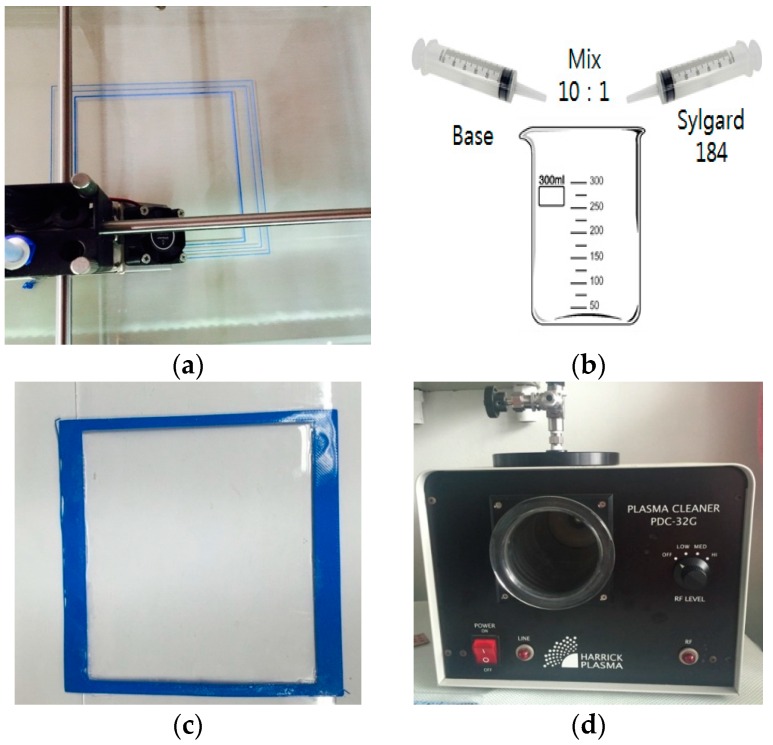
Polydimethylsiloxane (PDMS) fabrication process. (**a**) 3D printed molding of the PDMS substrate; (**b**) Mixing the base and curing agent; (**c**) Pouring liquid PDMS into the fabricated outline; (**d**) Plasma treatment processing.

Figure 4 shows the PDMS fabrication process. As shown in Figure 4a, the outline of the desired PDMS was first printed on a glass substrate using a 3D printer. The frame of the PDMS is fabricated using Ultimaker2+ 3D printer which is manufactured by Ultimaker B.V, in Geldermalsen, The Netherlands. Using 3D printing technology is easier and faster than conventional processes [24], and is currently widely used. The desired width (*W_s_*), length (*L_s_*), and thickness (*H_s_*) of the PDMS were 40, 50, and 1 mm, respectively. Having completed this, a liquid composition of a mixing base and a curing agent at a 10:1 ratio was prepared; a vacuum deaerator was used to remove the bubbles formed during the mixing process. The thick liquid composition can be cured at 25 °C for 48 h, or at 100 °C for 45 min. We performed heat curing at 80 °C for 30 min, using a hot plate. Next, for plasma treatment, we used the PDC-32G plasma cleaner from Harrick Plasma in U.S.A. Energy of plasma treatment for 20 s is 18 W.

### 3.2. Screen Printing

**Figure 5:**
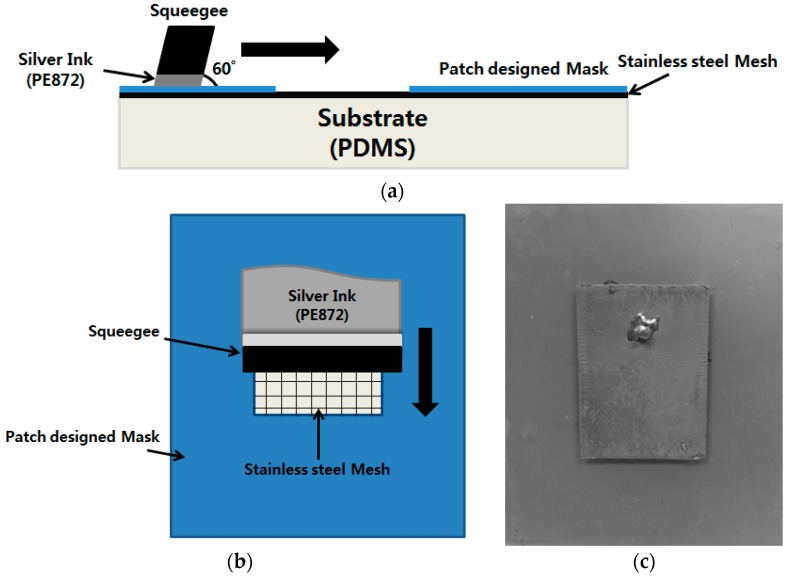
Silver screen printing process. (**a**) Screen printing process side view. (**b**) Screen printing process top view. (**c**) Picture of the fabricated prototype.

Figure 5 shows the silver screen printing process. As shown in Figure 5a,b, the conductive patterns of the top patch and bottom ground were screen printed on the PDMS using stretchable silver ink (Dupont PE872). We used the screen printer manufactured by Daeyoung Technology Co., (Bucheon, Korea). It provides 45–595 mm/s of printing speed, and a 60–90 degree of squeegee angle. The stainless steel mesh has 400 wire counts, and mesh tension is 150 N. We designed and applied a mask to the PDMS substrate, after which the silver ink was screen printed, using a squeegee. Figure 5c shows the completed device. On the top of the device is the printed patch sensor, and on the bottom is the printed ground. A curing process was applied to improve the conductivity of the screen printed surface. Thermal sintering method is used by a well-ventilated oven (ON-22GW) [25,26]. The curing was conducted at 100 °C for 30 min, in the oven. A hole was then drilled in the patch and the Sub-Miniature A (SMA) connector pin was inserted, after which the SMA connector’s inner conductor was attached to the sensor’s top side (patch), using silver conductive epoxy. In the same way, the SMA connector’s outer conductor was attached to the sensor’s bottom side (ground).

## 4. Measurement Results

**Figure 6:**
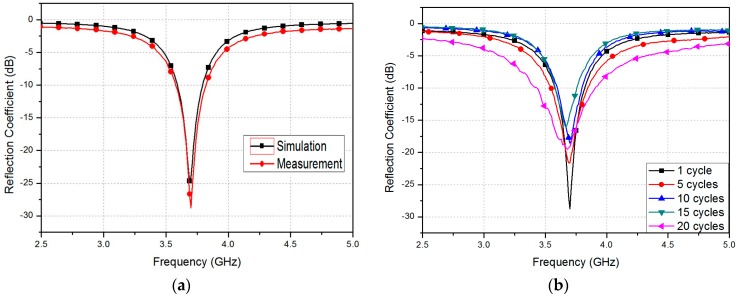
(**a**) Simulated and measured reflection coefficients of the proposed patch resonator before stretching; (**b**) Repeatability test of the fabricated strain sensor.

The fabricated RF strain sensor is shown in Figure 5c. The reflection coefficient of the proposed sensor is measured using Anritsu MS2038C vector network analyzer (Anritsu, Kanagawa, Japan). In Figure 6a, the measured reflection coefficient of the fabricated sample before stretching is compared to the simulated reflection coefficient in the same conditions. Initially, the unstretched patch sensor resonated at 3.7 GHz with a −28 dB reflection coefficient. The simulated and measured results show excellent agreement. In Figure 6b, the repeatability of the fabricated sensor is tested. Figure 6b shows the measured reflection coefficients at 1, 5, 10, 15, and 20 cycles. One cycle represents the un-stretch state after stretching the strain sensor. It is observed from Figure 6b that the resonant frequency is not changed until 10 cycles. The resonant frequency is slightly changed to 3.67 and 3.68 GHz at 15 and 20 cycles, respectively.

**Figure 7:**
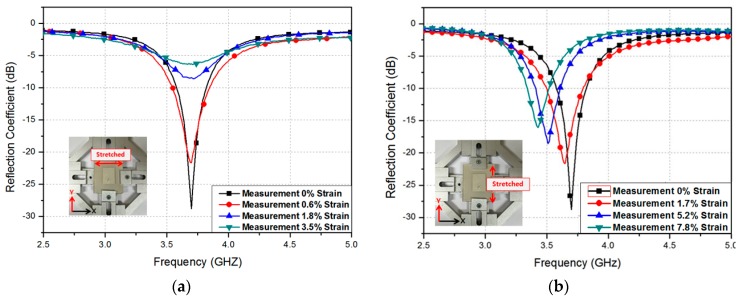
Measured reflection coefficients at different patch (**a**) widths and (**b**) lengths.

In addition, the reflection coefficient was measured at different strains. The fabricated patch prototype was stretched along the *x*- and *y*-directions, as shown in the inset of Figure 7. Figure 7a shows the measured reflection coefficients at different widths, when the patch is stretched along the *x*-direction. As expected from the simulation results in Figure 3a, the resonant frequency is not changed. The impedance changes, however, because the coaxial feeding hole is enlarged. Figure 7b shows the measured reflection coefficients at different lengths, when stretching the patch along the *y*-direction. As expected from the simulation results in Figure 3b, the resonant frequency changes from 3.7 GHz to 3.43 GHz when the patch is stretched from 0% to 7.82%. A maximum strain of 7.82%, which corresponds to 1.8 mm, was chosen, considering the PMDS mechanical strength.

**Figure 8:**
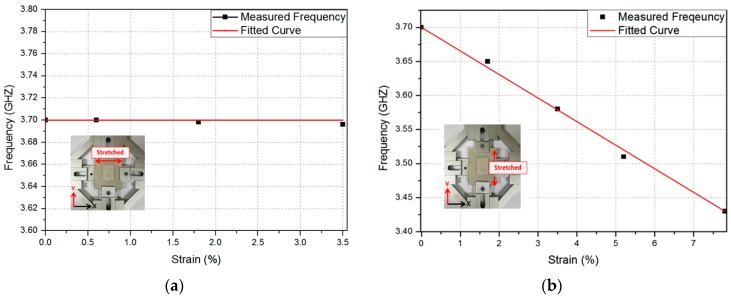
Relation between the resonant frequency and the strain along the (**a**) *x*-direction (width) and (**b**) *y*-direction (length).

The relation between the resonant frequency and the strain along the x-direction (width) and *y*-direction (length) is plotted in Figure 8a,b, respectively. The resonant frequency does not depend on the stretch along *x*-direction, as shown in Figure 8a, but is linearly proportional to the stretch along *y*-direction, as shown in Figure 8b. The fitting curve is given by *y* = −0.0343*x* + 3.7. Therefore, the sensitivity of the proposed RF strain sensor is 3.43 × 10^7^ Hz/%. The performance of the proposed strain sensor is compared with other strain sensors in Table 1. The proposed sensor shows higher maxim strain and strain gauge due to stretchable conductors and dielectric materials.

## 5. Conclusions

In this study, a screen-printed RF strain sensor using stretchable silver ink and PDMS was proposed. A rectangular patch resonator was designed and used as an RF strain sensor by considering the change in resonant frequency that results from stretches applied along the patch length dimension. The feasibility of this device was demonstrated using both simulation and measurements. Initially, the unstretched patch sensor resonated at 3.7 GHz with a −28 dB reflection coefficient. When the length of the patch sensor was increased by a 7.82% strain, the resonant frequency decreased from 3.7 GHz to 3.43 GHz. A sensitivity of 3.43 × 10^7^ Hz/% was therefore obtained. In contrast, the resonant frequency did not change when stretches were applied along the width dimension of the patch sensor. It can therefore be concluded that the proposed screen-printed RF patch resonator enables low-cost, high-volume fabrication of strain sensors.

## Figures and Tables

**Table 1:** Comparison table of proposed sensor and other RF strain sensors.

	This Work	[27]	[28]	[29]	[30]
Substrate	PDMS	Kapton tape	Si	Duroid 5880	Kapton
Conductive Material	Au	Au	Au	Cu	Cu/Al
Maximum Strain (%)	7.8	N/A	N/A	0.2	1
Strain Gauge ^1^ (%)	7.3	5.69	0.21	0.14	2.35
Resonant Frequency (GHz)	3.7	12.3	0.4742	5	3.62

^1^ Strain Gauge = Δf/f_0_ × 100 (%).
